# An *att* site-based recombination reporter system for genome engineering and synthetic DNA assembly

**DOI:** 10.1186/s12896-017-0382-1

**Published:** 2017-07-14

**Authors:** Michael J. Bland, Magaly Ducos-Galand, Marie-Eve Val, Didier Mazel

**Affiliations:** 1 Unité Plasticité du Génome Bactérien, Département Génomes et Génétique, Institut Pasteur, 75015 Paris, France; 2 UMR3525, Centre National de la Recherche Scientifique, 75015 Paris, France

**Keywords:** Site-specific recombination, Tyrosine recombinase, Serine recombinase, Genetic engineering

## Abstract

**Background:**

Direct manipulation of the genome is a widespread technique for genetic studies and synthetic biology applications. The tyrosine and serine site-specific recombination systems of bacteriophages HK022 and ΦC31 are widely used for stable directional exchange and relocation of DNA sequences, making them valuable tools in these contexts. We have developed site-specific recombination tools that allow the direct selection of recombination events by embedding the *attB* site from each system within the β-lactamase resistance coding sequence (*bla*).

**Results:**

The HK and ΦC31 tools were developed by placing the *attB* sites from each system into the signal peptide cleavage site coding sequence of *bla*. All possible open reading frames (ORFs) were inserted and tested for recombination efficiency and *bla* activity. Efficient recombination was observed for all tested ORFs (3 for HK, 6 for ΦC31) as shown through a cointegrate formation assay. The *bla* gene with the embedded *attB* site was functional for eight of the nine constructs tested.

**Conclusions:**

The HK/ΦC31 *att*-*bla* system offers a simple way to directly select recombination events, thus enhancing the use of site-specific recombination systems for carrying out precise, large-scale DNA manipulation, and adding useful tools to the genetics toolbox. We further show the power and flexibility of *bla* to be used as a reporter for recombination.

## Background

The ability to precisely and directly manipulate DNA is important for functional studies and the synthetic assembly of large genetic constructs. Site-specific recombinase (SSR) systems are widely used as tools to rearrange, insert, remove, and join DNA with virtually no upper limit in size. For biotechnology purposes, this can include the insertion of exogenous DNA into chromosomes, the fusing of DNA molecules, or the construction of synthetic gene networks [1]. The tyrosine (Y-rec) and serine (S-rec) recombination families are named for the catalytic residue of their respective integrase (Int) protein. Important members of the Y-rec family include the λ-like phage recombination systems, which include λ and the closely related phage HK022 (hereafter referred to as HK). The ΦC31 recombinase system is an important member of the S-rec family [2]. Both HK and ΦC31 systems comprise *attB*/*attP* attachment sites that serve as points of recombination, and the recombinases that catalyze recombination. In each family, DNA exchange requires host-encoded proteins for recombination that differ between systems. These systems are attractive due to their directionality and stability, and both systems are functional in prokaryotic and eukaryotic organisms [3–5].

Mechanistically, *attB* and *attP* integrative recombination forms *attL* and *attR* sites. The reverse *attL* x *attR* excisive reaction also requires Int as well as a recombination directionality factor (RDF), named Xis in the HK system and gp3 in the ΦC31 system [6], typically supplied *in trans* from a helper plasmid, a non-replicating DNA molecule, or as mRNA [7]. Structurally, HK and ΦC31 *att* sites differ in size, with the HK *attB* sites being generally shorter than the HK *attP* sites, 21 base pairs (bp) vs 234 bp [8, 9]; in addition, *attP* contains binding sites for Int and Xis along with host-encoded proteins Fis and IHF [8–11]. ΦC31 *attB* and *attP* sites are similar in size (~50 bp) and do not require additional proteins to carry out recombination [12].

**Fig. 1:**
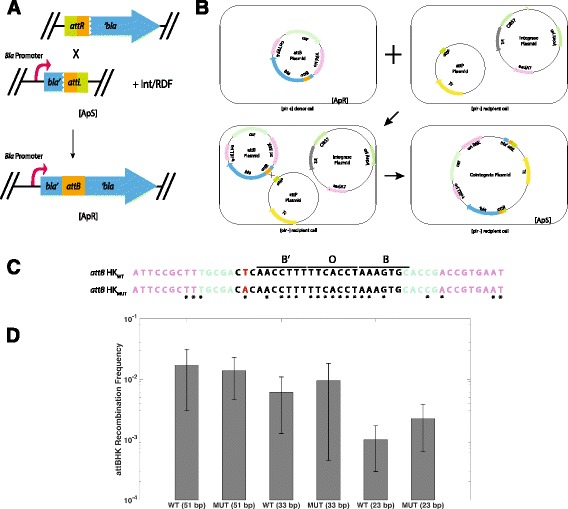
Schematic representation of *attB*-*bla* system and the conjugative assay used to test *att* sites. **a** In the selective tool, the *bla* gene is fragmented such that the 5′ promoter and signal sequence are associated with an *attL* site, and the partner *attR* is associated with the 3′ region. Each component is placed at separate loci, either on the genome or a plasmid, depending on the application. **b** Conjugation of the *attB* plasmid into a recipient strain containing the *attP* and integrase plasmids to form the *attR* and *attL* partners with *bla* gene fragments. **c** Sequence of the HK022 *attB* site. We tested *attB*_HK_ sites of three different lengths to avoid potential interference with *bla* function and protein export, 51 bp (*violet*), 33 bp (*teal*), and 23 bp (*black*). To increase the number of potential open reading frames, we introduced a T ➔ A nucleotide change into the *attB* sequence, indicated in red. The BOB’ core region is demonstrated by black lines. Stars indicate bases in common with *attP*_HK_. Recombination points flank the core O region. **d** Recombination results of *attB*_HK_ sequences. These six sequences were tested using a plasmid conjugation assay in a context independent of the *bla* gene [29]. This demonstrated that the introduced mutation did not interfere with recombination efficiency and the length of the *attB* site had a negative correlation with recombination frequency. As we wished to use a shorter sequence to avoid interfering with *bla* functionality following *attB* site insertion, we based our subsequent ORF constructions on the 23 bp mut form, despite the fact that it recombines at a lower frequency than the 51 and 33 bp wt sequences

The use of SSRs generally involves selecting the recombination event through the use of a marker gene within the inserted sequence whose presence or absence would indicate successful integration [1]. Genes can be activated following recombination through either removal of blocking DNA sequences or by bringing together physically separated congruous sequences, with the recombination site embedded within the gene or between the promoter and coding sequence. This approach has long been used with the popular *CRE/loxP* [13] and Flp/FRT [14] systems. The β-lactamase (*bla*) gene is an attractive marker, as it is a useful reporter gene for both pro- and eukaryotic applications [15]. Protein chimeras of β-lactamase demonstrate tolerance to exogenous peptide insertions [16], even for domains of unknown function [17]. A split gene reassembly approach using *bla* has also been developed to discover directed evolution-modified SSR enzymes capable of recombining designer sequences [18]. The *bla* signal peptide is an attractive region for peptide insertion [19], as insertions between the signal peptide sequence and the rest of the coding gene have minimal interference with protein function [20]. As we wished to expand the available molecular toolbox, we created a set of recombination reporters consisting of the *attB* of HK and ΦC31 inserted in frame with *bla*, allowing expression of the gene and enabling the direct selection of recombination events. The selective agent is not expressed when the *att* sites are in *attL* and *attR* form, as the reporter gene fragments are physically separated (Fig. 1a).

This approach has been used to explore the physical structure of the *E. coli* genome [21, 22]. Genome engineering of the two *Vibrio cholerae* chromosomes used this tool to understand the evolutionary and genetic implications of multi-chromosomal bacteria [23]. We have used HK recombination in tandem with the λ-*lacZ* system from [21] to exchange DNA between the two *V. cholerae* chromosomes in a recombination-mediated cassette exchange (RMCE), resulting in large-scale chromosomal rearrangements [23]. Because the *lacZ* reporter allows the observation of recombination events but not to select for them, we developed a reporter system for HK recombination based on antibiotic selection. We have used an HK *attB* site placed in-frame within the β-lactamase (*bla*) gene to carry out relocation of the *S10-spec-α* ribosomal locus in *V. cholerae* in order to study the consequences of essential gene positioning as it relates to dosage [24]. We further used HK-*bla* to carry out large-scale genome inversions around the origin region (*ori*) of *V. cholerae* chromosome one (Chr1) to shift the timing of the initiation of chromosome two (Chr2) replication relative to Chr1 in order to study the mechanisms involved in bacterial chromosome replication timing [25].

Here, we describe the construction and validation of HK-*bla* and a similar tool using the serine ΦC31 *att* system (ΦC31-*bla*). We placed *attB* sites from each system immediately downstream of the *bla* signal peptide coding sequence, which directs transport of β-lactamase to the periplasm and is removed in the mature protein. β-lactamase is generally tolerant of insertions into this region. When each system is present as *attL* and *attR* sites, they are associated with fragment sequences *bla’* (the 5′ region upstream of the cleavage site including the promoter and signal sequence) and *‘bla* (the 3′ region comprising the mature protein sequence), respectively (Fig. 1a). In addition, the cognate *att* site partners show high recombination frequencies without the presence of *bla*-resistant background from the fragmented *bla* gene. These systems are extremely useful due to their ability to directly select for recombination through resistance to β-lactam antibiotics. They also have the potential to be used within synthetic biology frameworks for constructing and precisely inserting large genetic assemblies, making them useful additions to the molecular biology toolbox for both synthetic and molecular applications.

## Results

### In-frame insertion of *attB*_HK_ sites within the ß-lactamase gene

The β-lactamase gene has a 23-amino acid (aa) signal peptide sequence for protein transmembrane transport that is cleaved during protein maturation [26]. We inserted the *attB* sequences in frame into the junction between the encoded signal sequence and the mature protein (Fig. 1a), as this region is tolerant to sequence insertions [19]. To avoid interfering with the β-lactamase coding sequence we took into account *attB* length and the amino acid sequence of the translated *att* sequence, so as to avoid frameshift or stop codon insertion.

### Recombination frequency in *attB*_HK_ sites decreases with size

**Fig. 2:**
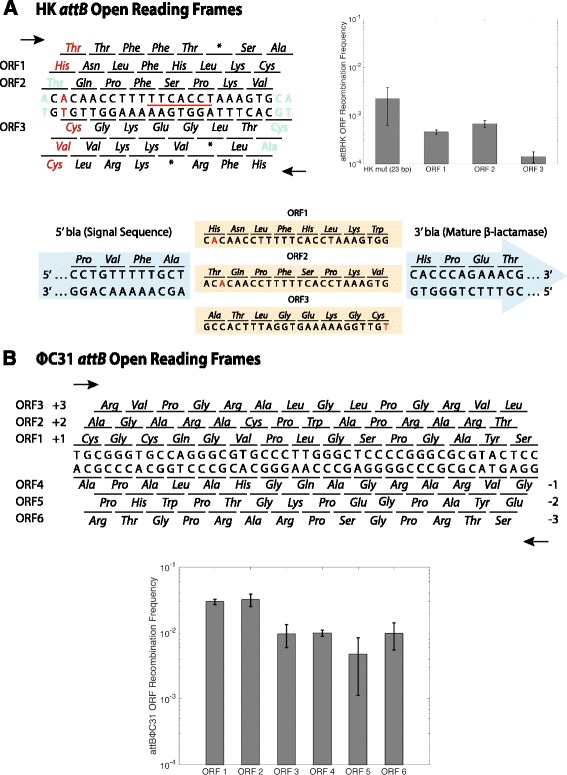
Sequences and recombination frequencies of HK and ΦC31 *attB* sites. The three ORFs for HK and the six ORFs for ΦC31 were inserted into *bla* and tested using the conjugation assay as in Fig. 1a. The six open reading frames of the 23 bp *attB* HK site are shown. As in Fig. 1, black nucleotides represent the 23 bp HK sequence, with the corresponding amino acids also in *black*. The *red* nucleotide shows the base changed from the original *attB*, with the resulting amino acid changes also shown in *red*. Nucleotides and amino acids in *teal* represent sequences flanking the 23 bp site. *Horizontal arrows* indicate the direction of transcription, and asterisks indicate a stop codon. For both **a** and **b**, the sequence of recombination exchange is indicated by a *horizontal red line*. As described in the text, three open reading frames did not have a stop codon and were able to be tested for *bla* insertion. The recombination frequencies of these open reading frames compared to the 23 bp HK *attB* site are shown in the bar graph. The open reading frames are also shown in context of the *bla* sequence flanking the insertion site. Note that to keep the *attB* site in frame with *bla*, nucleotides were added to either the 5′ or 3′ end of the site, which changed the expected amino acid residue for ORFs 1 and 3 compared to the original *attB*. The background colors highlighting the sequence correspond to Fig. 1a. The recombination frequencies of the different ORFs were compared using 1-way ANOVA followed by a Tukey-Kramer test. Each of the HK ORF recombination frequencies are significantly different (*p* < 0.05). **b**. None of the six ΦC31 ORFs encode a stop codon. ORFs 1 and 2 recombine at a higher rate than ORFs 3–6 (*p* < 0.001)

The *attB*_HK_ site comprises a 7 bp core, or overlap, (O) region where strand exchange occurs, and flanking B and B′ arm regions of 7 bp each that are recognized by Int monomers to form a synaptic complex, although sites shorter than this 21 bp have been shown to be functional but with low efficiency [27]. To allow recombination, the O region between *attB* and *attP* must perfectly overlap, and the arm regions must share similarity. Flanking the core minimal region, there are homologous nucleotides that may play an additional role in recombination efficiency [10, 28]. Insertion of *attB* into *bla* extends the gene and could affect either transport through the membrane or mature enzyme function. It is therefore necessary to test different open reading frames encoded by the *attB*_HK_ sequence to avoid unwanted interference with *bla*. The native *attB*_HK_ sequence encodes two open reading frames (ORFs) that do not have stop codons. As we wished to increase the potential sequences we could test within *bla*, we added a third potential ORF by mutating one bp just outside of the B′ region (Fig. 1c; Fig. 2a) [8, 27]. We compared these “mutant” *attB* sites to the “wild-type” sites to ensure there was no loss of recombination frequency (Fig. 1d).

The 23, 33, and 51 bp “wild type” and “mutant” *attB*_HK_ sequences were tested by placing them on the conditionally replicating conjugative plasmid pSW23T containing an *oriT*_RP4_ for plasmid conjugation and *oriV*_R6Kγ_ for π protein replication dependence (Fig. 1b); [29]. As these plasmids do not replicate in bacterial strains not expressing the π protein, conjugation into non-π expressing DH5α leads to plasmid loss unless *att* recombination occurs. The DH5α recipient strain houses plasmid pHK11Δamp, which has the *attP*_HK_ partner site, and pHK-Int, which expresses the HK integrase under control of the temperature-dependent CI857 promoter [30]. Following conjugation, recombination frequency was calculated by measuring the ratio of recovered colonies (representing co-integrates) over the number of recipient colonies [31]. Recombination frequencies were similar between the different sites, with only a 10-fold reduction in recombination observed for the 23 bp sites compared to the larger *attB* sites (Fig. 1c). As we wished to use a shorter sequence to avoid interfering with *bla* functionality following *attB* site insertion, we based our subsequent tests on the 23 bp *attB*_HK_ mutant form.

Placing a single nucleotide mutation in the 23 bp *attB*_HK_ site enables the use of three ORFs that would potentially allow *bla* function following their insertion into the gene (Fig. 2a). These ORFs were inserted separately into *bla* downstream of the signal sequence and cloned into pSW23T in a π + host. Following construction of these plasmids, we measured the ampicillin minimum inhibitory concentration (MIC) of each to test and measure *bla* function. All ORFs provided resistance to ampicillin at an MIC >256 μg/ml (Table 1). Recombination frequencies were then tested using the conjugation assay as above. The three HK ORF constructions demonstrated a wide range of recombination efficiencies, with the ORF 2 construct recombining at the highest level, and the ORF 3 construct recombining at the lowest (Fig. 2a). Thus, we used ORF 2 for the final construction of this tool.

**Table 1:** Minimum inhibitory concentration (MIC) of *attB*_HK_ and *attB*_ΦC31_ ORFs inserted into β-lactamase

Ampicillin Resistance of *bla*-*attB* ORFs	MIC (μg/ml)
HK022 ORF1	> 256
HK022 ORF2	> 256
HK022 ORF3	> 256
ΦC31 ORF1	> 256
ΦC31 ORF2	> 256
ΦC31 ORF3	> 256
ΦC31 ORF4	> 256
ΦC31 ORF5	6
ΦC31 ORF6	> 256

### ΦC31 *attB* x *attP* recombination is functional in all six ORFs

We designed *attB*_ΦC31_ sites for all six possible ORFs maintaining at least the minimal sequence necessary for recombination [32] and inserted them into *bla*. Ampicillin resistance and recombination frequency were determined as with the HK system. Five of six ORFs were found to provide MICs greater than 256 μg/ml, with the ORF 5 construction being the only sequence to interfere with β-lactamase function (MIC = 6 μg/ml - Table 1). ΦC31 pSW23T-bla plasmids were conjugated into a DH5α strain harboring plasmids pΦC31-Int and pΦC31-attP. All six ORFs were able to recombine successfully, with ORF constructions 1 and 2 recombining at a higher rate, on the order of 10^−2^, than ORFs 3–6, which recombined at an average rate of 10^−3^ (Fig. 2b). We found this difference to be significant using a 1-way ANOVA (*p* < 0.001) followed by a post-hoc Tukey-Kramer test (*p* < 0.001). Additionally, all six ΦC31 ORF constructions recombined at a higher rate than HK ORFs 1–3 (Fig. 2).

## Discussion

In this study, we describe the construction of two site-specific recombination tools useful for DNA manipulation applications. The utility of this *attB*-*bla* tool is based on its incorporation of the widely used HK and ΦC31 recombination systems. In the case of HK, the removal of sequences flanking the BOB’ core region reduced *attB* x *attP* recombination. This reduction could be due to the removal of bases outside of the *attB* core that have homology with the *attP* sequence, which may act to stabilize the *attB*/*attP* complex. However, obtaining the highest possible recombination frequency was not critical for the design of this system, as our main concern was β-lactamase function following insertion of the *att* sites into the *bla* coding frame.

In directly comparing the two systems, the ΦC31 site appears to recombine at a similar frequency to the 51 bp HK sites and the 23 bp HK ORFs incorporated into *bla* have a lower recombination frequency (Fig. 2). This decrease is likely due to the reduction of size of the *attB*_HK_ site, as the recombination frequencies for the smaller HK site tested independently of *bla* insertion are not different from the frequencies obtained when they are embedded in *bla* (Fig. 1). Reported differences between recombination systems in the literature may result from differences in protocols and practices. A recent review of ΦC31 found a wide range of reported recombination frequencies for this recombinase [33]. To our knowledge, the only information comparing HK and ΦC31 recombination frequencies reports HK recombining at a higher frequency than ΦC31 [34]. However, this study used a clonetegration technique where constructs were recombined into native *att* sites on either the *E. coli* chromosome for *attP*_HK_ or *Salmonella typhimurium* for *attP*_ΦC31_.

While testing *bla* expression with inserted ORFs, we observed that ΦC31 ORF 5 interfered with *bla* expression, while ΦC31 recombination was not affected (Table 1, Fig. 2b). The *bla* gene used for our system originates from pBR322 and belongs to the TEM-1 class of β-lactamases. The signal sequence is recognized by the Sec export pathway that transports unfolded proteins across the cytoplasmic membrane [26, 35]. DNA secondary structures could be a source of transcription interference, as ORF 5 forms a 30 bp hairpin (∆G at 37 °C = −9.09 kcal/mol). However, hairpins are formed in all 6 ORFs at similar ∆G, making it unlikely that this factor alone prevents *bla* expression. At the translation level, the overall charge of the first 5 amino acids following the signal sequence can influence cleavage and cross-membrane transport, as they generally have an overall negative charge [36]. For ORF 5, the overall negative charge of this region is +2. Again, however, this is unlikely to explain the loss of *bla* expression, as only ORF 1 has an overall negative charge, at −1. The amino acids in the 1 and 2 position after the cleavage site can also influence protein function [37, 38]. For ORF 5, the first two amino acids are glycine and serine. Analysis of 307 proteins from the SPdb database [39] found that in Gram-negative bacteria, glycine occurs in the 1st position in 6.19% of proteins, and serine appears in the 2nd position in 5.54% of proteins. [40]. Additionally, two of the 307 Gram-negative proteins analyzed in this study begin with glycine-serine. Thus it is unlikely that the first two residues of the ORF 5 sequence alone interfere with protein transport. More experimental and analytical work is needed to determine the source of *bla* expression interference.

The high tolerance of *bla* to in-frame DNA sequence insertion downstream of the *bla* promoter and leader peptide sequence allows for further modifications of this system through insertion of potentially large ORFs. This approach has already been proposed as an “ORF-trap” to capture DNA encoding protein fragments [41]. Indeed, large ORFs in frame with *bla* may not greatly reduce β-lactamase function, although export to the periplasm can be inhibited [42]. Additionally, as *attB* and *attP* site reactivity can be modified through mutations to their respective core sequences, variable non-reacting “synthetic” *att* sites can be designed for sequential introduction into the bacterial chromosome [43].

Integration of exogenous DNA sequences into genomes by SSRs generally involves the recombination of an *attP* site on the inserted sequence with an endogenous chromosomal *attB* or pseudo-*attB* site [1]. The use of genome editing technologies allows the insertion of recombination sites that differ from native sites in location and sequence. Native *att* sites may be located in undesirable regions of the genome, for example, in an active gene locus, or a locus subject to silencing. Additionally, dosing effects can be observed in bacterial species dependent on a gene’s location in the chromosome [24]. Engineering *att* site recognition by Int proteins allows the creation of semi-synthetic partner sites [27, 43]. This would avoid recombination with other native *att* sites, and could allow rapid construction of synthetic gene networks. The addition of FRT sites flanking the *bla-attB* cassette would further allow for removal of the resistance selection marker gene. Similarly, gene-editing technologies could allow the targeted insertion of *att* sites to serve as landing pads for insertion. In this way, the *bla’-attL* sequence from our system can be inserted into a genome, into which a sequence containing the partner *attR-‘bla* can be inserted through *attL* x *attR* recombination. This framework has already been proposed for the construction and insertion of metabolic networks into eukaryotic cell lines [44]. Our system adds the advantage of avoiding marker expression until recombination, making it versatile for synthetic applications as well as genome-scale engineering.

## Conclusions

We describe here the construction of new tools based on two different site-specific recombination systems, the tyrosine recombinase HK, and the serine recombinase ΦC31. Recombination for each system is reported based on the reconstitution of the *bla* ampicillin resistance gene, providing resistance to β-lactam antibiotics as a selective agent. Both HK-*bla* and ΦC31-*bla* are useful for selecting recombination events in a genomic context due to a high rate of recombination frequency, directionality based on the recombination proteins supplied *in trans*, and the ability to carry out in vivo genomic rearrangements. We have previously used this tool in our lab to carry out large-scale reorganization of the *V. cholerae* chromosomes to study the importance of chromosome size in multi-chromosomal bacteria [23], the relevance of genome position and chromosome location for gene dosage and its evolutionary importance [24], and the timing of *V. cholerae* chromosome replication [25]. The importance of these tools lie in their capacity to exist simultaneously in the cell at two separate loci without expression of the marker gene until expression of the recombination proteins is induced.

## Methods

### Bacterial strains and media

Bacterial strains used in this study are described in Table 2. All strains were grown in lysogeny broth (LB) medium at 30 °C, 37 °C, or 42 °C depending on plasmid temperature-sensitivity. Antibiotic and nutritional supplement concentrations were as follows: ampicillin (Ap): 100 μg/ml, carbenicillin (Carb): 100 μg/ml, kanamycin (Km): 25 μg/ml, chloramphenicol, (Cm): 25 μg/ml, tetracyclin (Tc): 15 μg/ml, spectinomycin (Sp): 100 μg/ml, erythromycin (Em): 20 μg/ml, with nutritional supplements diaminopimelic acid (DAP): 300 μM, and thymine (dT): 300 μM.

**Table 2:** Bacterial strains used in this study

*E. coli*		
Name	Genotype	Reference/Source
β2163	(F^−^) RP4–2-Tc::Mu Δ*dapA*::(*erm-pir*) [Km^R^ Em^R^]	[29]
π1	DH5αΔthyA::(erm-pir116) [Em^R^]	[29]
MFD*pir*	MG1655 RP4–2-TC::[Mu1::*aac(3)IV-*Δ*aphA-*Δ*nic*35-ΔMu2::*zeo*] Δ*dapA::*(*erm-pir*)Δ*recA*[Apra^R^ Zeo^R^Erm^R^]	[47]
PGB-8557	DH5α strain containing plasmids pHKΔ-Amp and pHK-Int [Tc^R^ Sp^R^]	this study
PGB-E274	DH5α strain containing plasmids pΦC31-attP and pΦC31-Int [Tc^R^ Sp^R^]	this study
One Shot ® Top10	F- *mcrA* Δ(*mrr-hsd*RMS-*mcr*BC) Φ80*lac*ZΔM15 Δ *lac*X74 *rec*A1 *ara*D139 Δ(*araleu*)7697 *gal*U *gal*K *rps*L (StrR) *end*A1 *nup*G	ThermoFisher Scientific

### Cloning

Basic cloning steps were performed using the following tools and appropriate protocols: for DNA purification, a QIAquick PCR purification kit (QIAGEN) was used. Plasmid minipreps were performed using the GeneJET Plasmid Miniprep kit (Life Technologies). All PCR reactions for plasmid construction were performed using the Phusion High-Fidelity PCR Master Mix (Life Technologies), and all diagnostic PCR reactions were performed using DreamTaq DNA Polymerase (Life Technologies). Oligonucleotides were synthesized by Sigma-Aldrich and Eurofins Genomics. Oligonucleotides were phosphorylated by T4 polynucleotide kinase (NEB). DNA was sequenced by GATC Biotech and Eurofins Genomics.

### Construction of plasmids

Insertion of *attB s*equences into pSW23T was performed by annealing phosphorylated oligos containing the respective *att* sequence with overhangs overlapping with *Bam*HI and *Eco*RI restriction sites, followed by cloning of these sequences into the pSW23T fragment. Insertion of *attP* sequences into pHK11-Amp was similarly performed. The various *attB* ORFs for both HK and ΦC31 were inserted into the β-lactamase (*bla*) by overlapping PCR, in which the 5′ region of *bla* was amplified from pMP58 using oligos MV26 and the appropriate reverse *attB* oligo, and the 3′ *bla* region amplified using a forward *attB* oligo and JB13. These products were gel purified and co-amplified using oligos MV26 – JB13 to form a DNA fragment containing *bla* with the inserted *attB*. This product was digested with EagI and EcoRI and cloned into pSW23T and transformed into MFDpir. The pMP58 *bla* gene comes from pUC19.

To make plasmid pPhiC31-Int, we first deleted the XbaI site in pZJ7 (a kind gift of Jia Zhao and Sean Colloms) by digestion with SpeI-XbaI followed by religation to make plasmid pZJ7ΔXbaI. The ΦC31 integrase gene was amplified using oligos PhiC31 IntF and PhiC31 IntR. The pHK-Int backbone was amplified using oligos JB485 and JB486. These oligos produce DNA fragments with overlapping ends, which were then joined by Gibson assembly [45]. Plasmids used in this study are listed in Table 3 and oligonucleotides in Table 4.

**Table 3:** Plasmids used in this study

Name	Description	Reference/Source
pSW23T	pSW23::oriTRP4; [Cm^R^]; oriVR6K	[29]
pSU38Δ	orip15A [Km^R^]	[48]
pHK-Int	pGB2ts::cI857-λ-P_R_-HKInt, [Sp^R^]	[30]
pHK11-Amp	pLDR11::attP_HK, [Ap^R^,Tc^R^]	[30]
pSC101	pSC101ts, repA [Tc^R^]	[49]
pUC19	oriColE1, lacZα [Ap^R^]	[50]
pBAD43	oripSC101, PBAD::MCS,[Sp^R^]	[51]
pHK11Δamp	pHK11-Amp::attP_HK,ΔAmp, [Tc^R^]	this study
pMP96	pSC101ts::*c*I857-λ-P_R_-(HK_Xis_-HK_Int_ λ_Xis_-λ_Int_), [Sp^R^]	[23]
pMP58	pSC101ts::oriTRP4;repA, [Cm^R^,Ap^R^]	this study
pMDG1	pMP58;bla::attB_HK,[Ap^R^,Cm^R^]	this study
pMDG2	pSW23T::bla::attB_HK from pMDG1	this study
pMDG3	α/pSU38::attR_HK, [Ap^R^]	this study
pMDG4	pSW23T::attL_HK, [Cm^R^]	this study
pMJM1	pSW23T::attB_HKwt, [Cm^R^]	this study
pMJM2	pSW23T::attL_HKmut, [Cm^R^]	this study
pMJM3	pSW23T::attL_HK40, [Cm^R^]	this study
pMJM4	pSW23T::attL_HK30, [Cm^R^]	this study
pJB6	pSU38Δ::attR_HK-attL_λ, [Ap^R^]	this study
pJB7	pSW23T::attR_HK-attL_λ, [Cm^R^]	this study
pJB8	pBAD43::HK_Xis_-HK_Int_ λ_Xis_-λ_Int_, [Sp^R^]	this study
pZJ7	pBAD33::ΦC31Int, [Cm^R^]	J. Zhao and S. Colloms
pZJ7ΔXbaI	pZJ7 with SpeI – XbaI fragment deleted	this study
pPhiC31-Int	pGB2ts::cI857-λ-PR-ΦC31Int, [Sp^R^]	this study
pPhiC31-attP	pHK11Δamp::attP_ΦC31, [Tc^R^]	this study

**Table 4:** Oligonucleotides used in this study

Oligonucleotide	Sequence 5′ – 3′
PhiC31 Int F	ATGTACTAATCTAGAGAAGAGGATCAGAAATGGACACGTACGCGGGTGC
PhiC31 Int R	CAAGCTTGCATGCCTGCAGG
JB13	AGCGGGTGTTCCTTCTTCACTG
JB485	TCTTCTCTAGATTAGTACATGCAACCA
JB486	CGACTAGAGTCGACCTGCAGCCAAGCTTAGTAAAGCCCTC
MV26	ACGGCTGACATGGGAATTGC
MV143	CCTCTTACGTGCCGATCAACGTCTC
MV145	GCTGGTGATTCCGCTTTGCGACTCAACCTTTTTCACCTAAAGTGCACCGACCGTGA
MV146	ACATCAGCGATCACCTGGCAGAC
attBHKwtERI	AATTCCGCTTTGCGACTCAACCTTTTTCACCTAAAGTGCACCGACCGTGAATG
attBHKwtREV	GATCCATTCACGGTCGGTGCACTTTAGGTGAAAAAGGTTGAGTCGCAAAGCGG
attBHKmutERI	AATTCCGCTTTGCGACACAACCTTTTTCACCTAAAGTGCACCGACCGTGAATG
attBHKmutREV	GATCCATTCACGGTCGGTGCACTTTAGGTGAAAAAGGTTGTGTCGCAAAGCGG
40wtERI	AATTCTGCGACTCAACCTTTTTCACCTAAAGTGCACCG
40wtREV	GATCCCGGTGCACTTTAGGTGAAAAAGGTTGAGTCGCAG
40attBHKmutERI	AATTCTGCGACACAACCTTTTTCACCTAAAGTGCACCG
40attBHKmutREV	GATCCCGGTGCACTTTAGGTGAAAAAGGTTGTGTCGCAG
30wtERI	AATTCTCAACCTTTTTCACCTAAAGTG
30wtREV	GATCCACTTTAGGTGAAAAAGGTTGAG
30attBHKmutERI	AATTCACAACCTTTTTCACCTAAAGTG
30attBHKmutREV	GATCCACTTTAGGTGAAAAAGGTTGTG
30attBHKamp2ORF1min	TTTGCTCACAACCTTTTTCACCTAAAGTGGCACCCAGAAACGCTGGTGAAAGTAAAAGATGCTGAAGATCAGTT
30attBHKamp1ORF1min	CTTTCACCAGCGTTTCTGGGTGCCACTTTAGGTGAAAAAGGTTGTGAGCAAAAACAGGAAGGCAAAATGCCGC
30attBHKamp2ORF2min	TTTGCTACACAACCTTTTTCACCTAAAGTGCACCCAGAAACGCTGGTGAAAGTAAAAGATGCTGAAGATCAGTT
30attBHKamp1ORF2min	CTTTCACCAGCGTTTCTGGGTGCACTTTAGGTGAAAAAGGTTGTGTAGCAAAAACAGGAAGGCAAAATGCCGC
30attBHKamp2ORF3min	TTTGCTGCCACTTTAGGTGAAAAAGGTTGTCACCCAGAAACGCTGGTGAAAGTAAAAGATGCTGAAGATCAGTT
30attBHKamp1ORF3min	CTTTCACCAGCGTTTCTGGGTGACAACCTTTTTCACCTAAAGTGGCAGCAAAAACAGGAAGGCAAAATGCCGC
phiC31 ORF1 F	TTTGCTTGCGGGTGCCAGGGCGTGCCCTTGGGCTCCCCGGGCGCGTACTCCCACCCAGAAACGCTGGTGAAAG
phiC31 ORF2 F	TTTGCTGCGGGTGCCAGGGCGTGCCCTTGGGCTCCCCGGGCGCGTACTCCCCACCCAGAAACGCTGGTGAAAG
phiC31 ORF3 F	TTTGCTCGGGTGCCAGGGCGTGCCCTTGGGCTCCCCGGGCGCGTACTCCCCCACCCAGAAACGCTGGTGAAAG
phiC31 ORF4 F	TTTGCTGGAGTACGCGCCCGGGGAGCCCAAGGGCACGCCCTGGCACCCGCACACCCAGAAACGCTGGTGAAAG
phiC31 ORF5 F	TTTGCTGGGAGTACGCGCCCGGGGAGCCCAAGGGCACGCCCTGGCACCCGCCACCCAGAAACGCTGGTGAAAG
phiC31 ORF6 F	TTTGCTGGGGAGTACGCGCCCGGGGAGCCCAAGGGCACGCCCTGGCACCCGCACCCAGAAACGCTGGTGAAAG
phiC31 ORF1 R	CTTTCACCAGCGTTTCTGGGTGGGAGTACGCGCCCGGGGAGCCCAAGGGCACGCCCTGGCACCCGCAAGCAAAAACAGGAAGGCAAAATG
phiC31 ORF2 R	CTTTCACCAGCGTTTCTGGGTGGGGAGTACGCGCCCGGGGAGCCCAAGGGCACGCCCTGGCACCCGCAGCAAAAACAGGAAGGCAAAATG
phiC31 ORF3 R	CTTTCACCAGCGTTTCTGGGTGGGGGAGTACGCGCCCGGGGAGCCCAAGGGCACGCCCTGGCACCCGAGCAAAAACAGGAAGGCAAAATG
phiC31 ORF4 R	CTTTCACCAGCGTTTCTGGGTGTGCGGGTGCCAGGGCGTGCCCTTGGGCTCCCCGGGCGCGTACTCCAGCAAAAACAGGAAGGCAAAATG
phiC31 ORF5 R	CTTTCACCAGCGTTTCTGGGTGGCGGGTGCCAGGGCGTGCCCTTGGGCTCCCCGGGCGCGTACTCCCAGCAAAAACAGGAAGGCAAAATG
phiC31 ORF6 R	CTTTCACCAGCGTTTCTGGGTGCGGGTGCCAGGGCGTGCCCTTGGGCTCCCCGGGCGCGTACTCCCCAGCAAAAACAGGAAGGCAAAATG

### Recombination assay

Recombination frequencies were tested by performing a conjugation assay in which the plasmid pSW23T containing the *oriT*_RP4_ transfer region and *oriV*_R6K_ π-controlled replication origin were transferred from the π+/DAP- donor strain MFDpir to a recipient strain containing an *attP* plasmid and a helper plasmid expressing the appropriate integrase gene under control of the temperature-sensitive CI857 promoter. Prior to conjugation, strains were diluted 1/100 from an overnight starter culture and grown to OD_600_ = 0.3. Conjugations were performed by two techniques: for the *attB* HK_WT/MUT_ strains, 0.5 ml of donor was mixed with 4.5 ml of recipient and applied to a 0.45 μm filter (Millipore) by vacuum-filtration through a glass column. The *attB* ORF insertions into *bla* were performed by mixing 0.2 ml of donor with 1.8 ml of recipient, and following centrifugation at 6000 RPM for 5 min, ~1.8 ml of supernatant was removed, the pellet resuspended in the remaining liquid media, and similarly placed onto a 0.45 μm filter. For both techniques, the filters were then incubated on an LB-DAP plate for approx. 16 h prior to resuspension and plating. Recombinants were recovered by selecting for Cm resistance in DAP-free media, and recombination frequencies were measured as the ratio of recovered recombinants over donor CFUs. Each *att* site was tested three times.

### Minimum inhibitory concentration (MIC)

The MICs of *E. coli* strains containing plasmids with either *attB* inserted into *bla*, or *bla* fragments associated with *attL* and *attR* were performed by plating and aspirating 2 ml of a 1/100 dilution of an overnight culture onto an LB/DAP agar petri dish. An Etest (bioMérieux) ampicillin antibiotic strip was placed onto the plate and incubated overnight at 37 °C, and the level of antibiotic resistance was scored the following day.

### Data analysis

Recombination frequencies were analyzed for statistical significance using MATLAB software (The MathWorks, Inc., Natick, MA). 1 and 2-way analysis of variance (ANOVA) tests were performed using the anova1 and anova2 functions. Tukey-Kramer post-hoc tests were performed using the multcompare function.

### DNA folding and protein structure analysis

Secondary DNA structures were analyzed using the mfold software [46]. Protein residue charges were calculated by counting negatively charged residues Asp and Glu as −1, and positively charged His, Lys, and Arg as +1.

## Abbreviations

Bla: β-lactamase
HK022: HK
Int: Integrase
MIC: Minimum inhibitory concentration
O region: Overlap region
ORF: Open reading frame
RDF: Recombination directionality factor
S-rec: Serine recombinase
SSR: Site-specific recombinase
Y-rec: Tyrosine recombinase
